# Cataracts in Havanese: genome wide association study reveals two loci associated with posterior polar cataract

**DOI:** 10.1186/s40575-023-00127-y

**Published:** 2023-04-28

**Authors:** Kim K. L. Bellamy, Frode Lingaas

**Affiliations:** 1 The Norwegian Kennel Club, Oslo, Norway; 2 Department of Preclinical Sciences and Pathology, Faculty of Veterinary Medicine, Norwegian University of Life Sciences, Ås, Norway

**Keywords:** GWAS, Cataract, Havanese, ECVO, *ANO3*, *FOXP1*, *RYBP*, *LGR4*, *ANK3*, *PCLAF*

## Abstract

**Background:**

Cataract is considered an important health issue in Havanese, and studies indicate a breed predisposition. Possible consequences of cataracts include lens induced uveitis, reduced eyesight, and blindness in severe cases. Reducing the prevalence of cataracts could therefore improve health and welfare significantly. The most frequently diagnosed forms of cataract in Havanese are cortical- and anterior suture line cataract, but cases of posterior polar cataract are also regularly reported. Out of the three, posterior polar- and cortical cataracts are considered the most clinically relevant.

**Results:**

We performed a genome wide association study that included 57 controls and 27 + 23 + 7 cases of cortical-, anterior suture line- and posterior polar cataract, respectively. An association analysis using a mixed linear model, revealed two SNPs on CFA20 (BICF2S23632983, *p* = 7.2e-09) and CFA21 (BICF2G630640490, *p* = 3.3e-09), that were significantly associated with posterior polar cataract, both of which are linked to relevant candidate genes. The results suggest that the two variants are linked to alleles with large effects on posterior polar cataract formation, possibly in a dominant fashion, and identifies regions that should be subject to further sequencing.

Promising regions on CFA4 and CF30 were also identified in the association analysis of cortical cataract. The top SNPs on each chromosome, chr4_12164500 (*p* = 4.3e-06) and chr30_28836339 (*p* = 5.6e-06), are located within, or in immediate proximity to, potential cataract candidate genes.

The study shows that age at examination is strongly associated with sensitivity of cataract screening. Havanese in Norway are on average 3.4 years old when eye examinations are performed: an age where most dogs that are genetically at risk have not yet developed clinically observable changes. Increasing the average age of breeding animals could increase accuracy of selection, leading to improved health.

**Conclusions:**

The study identified two loci, on CFA20 and CFA21, that were significantly associated with posterior polar cataract in Havanese. SNPs that showed putative association with cortical cataracts, were observed on CFA4 and CFA30. All the top SNPs are located in close proximity to cataract candidate genes. The study also show that sensitivity of cataract screening is highly dependent on age at examination.

**Supplementary Information:**

The online version contains supplementary material available at 10.1186/s40575-023-00127-y.

## Background

Cataract is considered an important health challenge in the Havanese dog breed [1], and is characterized by opacities of the lens [2]. Approximately 5% of ECVO eye examinations of Havanese in Norway result in a cataract diagnosis [1, 3], and in a study from North America, Havanese were the second most frequently diagnosed breed, with an estimated prevalence of 11.57% [4]. Closely related breeds, like the bichon frisé, Maltese and Bolognese, are also predisposed [4–6].

Cataracts are defined by lens opacities [2]. The lens is normally a dehydrated structure, which consists primarily of proteins, and the structure and organisation of these are crucial to maintain lens transparency. Swelling of lens fibers or disruption of proteins, for example because of fluid absorption, will result in light scatter and an opaque lens. Cataracts must not be confused with the normal increase in nuclear density in older dogs, called nuclear sclerosis [7].

Cataracts in dogs are often classified based on age at onset, localization in the lens and degree of opacification [2, 7]. The most commonly reported forms of cataracts in Havanese in Norway, are cortical-, anterior suture line-, and posterior polar cataracts [3], which is in accordance with literature [7]. Cortical cataract is also the most frequently diagnosed type of cataract in the closely related breed bichon frise [6]. The clinical significance is dependent on the localization, density and extent of the opacity, as well as the rate of progression, which means posterior polar- and cortical cataracts are generally considered more clinically relevant than anterior suture line cataract [8].

Cataracts can cause reduced eyesight or blindness, depending on the extent of the opacities. In addition to its effect on eyesight, cataracts can also lead to lens induced uveitis caused by leakage of lens proteins [2, 7]. In cases where it is necessary to treat the cataract, the only definite therapy in dogs is surgical removal of the entire lens. The surgery is invasive, requires substantial follow-up by the veterinarian and owner, and is associated with risk of postoperative complications. Postoperative complications can include postoperative uveitis, glaucoma, retinal detachment, or phthisis bulbus [2, 7].

Causative mutations for cataracts have been identified in some dog breeds. In the wirehaired pointing griffon, a mutation in the FYCO1 gene, which encodes a protein that is important for lens autophagy, was identified as the likely cause of a form of juvenile cataracts [9]. *FYCO1* is also associated with many forms of cataracts in humans [10–12]. Mutations in the HSF4 gene cause a recessive form of cataract in Staffordshire bull terriers and Boston terriers, a dominant form of cataract in Australian Shepherds [13, 14], as well as several forms of cataracts in humans [15, 16]. In many breeds, studies have failed to identify causative variants [17–20], even though there is evidence of a strong genetic influence on the trait [6, 18, 21].

Since April 2016, eye examinations for both parents within a maximum of one year prior to breeding, has been a requirement for registration of Havanese puppies in The Norwegian Kennel Club [22]. However, yearly eye examinations in breeding animals have been customary for Havanese breeders prior to 2016 as well, due to breed club recommendations [23]. We have previously shown that 21% of Havanese that are registered in the Norwegian Kennel Club database have their eyes examined at least once in their lifetime, and that at least one puppy is examined in half of all registered Havanese litters [24].

Despite almost two decades of good compliance to the screening program and using only unaffected individuals for breeding, the prevalence of cataracts in Havanese in Norway has been stable [1]. We hypothesized that the main reason for this may be the late onset of disease. Many affected individuals have already had several litters at the time of diagnosis, which means the true selection pressure is very low. In addition, some sources indicate that the mode of inheritance could be recessive [7], which would mean that most risk alleles in the population are hidden in heterozygotes and that excluding affected dogs from breeding would only remove a minor proportion of risk alleles [25]. A better understanding of the genetic background of cataracts in Havanese, and ideally the identification of causative mutations, would be valuable to improve efficacy of the breeding program.

The aim of this study was to explore the genetic background of cataracts in Havanese. To identify genomic regions that may be important in cataract development and could be subject to further analysis and fine mapping, we conducted a genome wide association study (GWAS). We also examined age of onset and effect of age on test sensitivity, to estimate a reasonable minimum age for controls, for use in both genetic studies and breeding strategies.

## Results

### Population data analysis

#### No obvious changes in prevalence from 2003–2022

Preliminary investigations had indicated that the current screening program and breeding policy had not successfully reduced the prevalence of cataracts in Havanese. Our results confirm that there has been no decline in observed prevalence of cataracts during the last two decades (Table 1 and 2).

**Table 1:** Eye screening results for Havanese, year 2003–2022, from the Norwegian Kennel Club database

*Year of examination*	*N examinations*	*Affected*	*Unaffected*	*Suspected*	*Prevalence (95% CI)*
*2003–2007*	104	1	103	0	1.0% (0–2.8%)
*2008–2012*	545	25	509	11	4.6% (2.8–6.3%)
*2013–2017*	800	38	739	23	4.8% (3.3–6.2%)
*2018–2022*	1132	66	1033	33	5.8% (4.5–7.2%)
*Total*	**2581**	**130**	**2384**	**67**	**5.0% (4.2–5.8%)**

**Table 2:** Eye screening results for Havanese, year 2003–2022, from the Norwegian Kennel Club database

*Year of examination*	*Cortical*	*Post polar*	*Ant.sut.l*
*2003–2007*	1	0	0
*2008–2012*	12	3	9
*2013–2017*	21	3	15
*2018–2022*	20	4	31
*Total*	**54**	**10**	**55**

#### Age at examination

We found that the average age at examination (AAE) was 3.4 years (*SD* = 1.9), and that only 7.7% of examinations were performed in dogs that were 7 years or older (AAE ≥ 7). The majority of Havanese that underwent an eye examination were only examined once (764/1409).

#### Age at onset

Average age at onset (AAO) for all cataracts in Havanese was 4.8 years (*SD* = 1.4). Average AAO for the different forms of cataracts: cortical cataracts (*M* = 4.5, *SD* = 1.6), anterior suture line cataracts (*M* = 5.0, *SD* = 1.4), and posterior polar cataracts (*M* = 3.0, *SD* = 1.4), were not significantly different (cortical–ant.sut.l *t*(34) = 0.99, *p* = 0.33; cortical – post.pol *t*(14) = 1.25, *p* = 0.23; ant.sut.l – post.pol *t*(22) = 1.93, *p* = 0.06).

#### Effect of AAE on observed prevalence

**Fig. 1:**
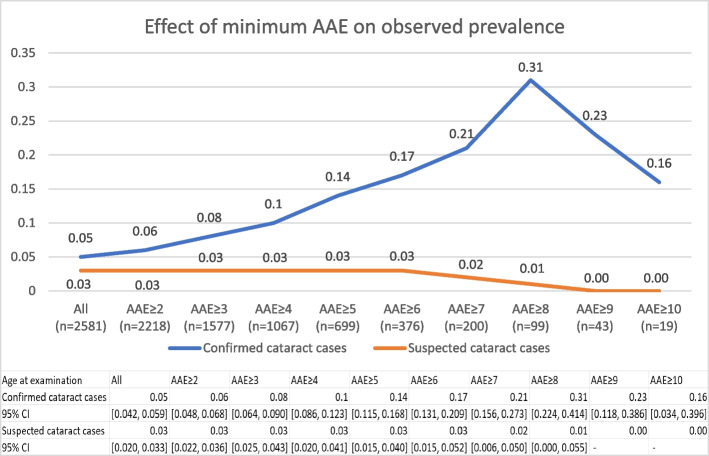
The proportion of examinations that resulted in a cataract diagnosis was positively correlated with minimum age at examination. The total number of eye certificates prior to filtering on AAE was 2,581. The number of remaining records after filtering on minimum AAE, is shown in brackets

We examined how the observed prevalence of cataracts would be affected by removal of results from young dogs, by applying different cut-off points for minimum AAE to the complete dataset of Havanese eye certificates (including repeat examinations). Between AAE ≥ 1 and AAE ≥ 8, observed prevalence was positively correlated with AAE (*r*(6) = 0.95, *p* = 0.0003). The prevalence was highest in the AAE ≥ 8-group, in which almost one third of the dogs were diagnosed with cataracts. The frequency of suspected cataract cases was negatively correlated with AAE (*r*(8) = -0.87, *p* = 0.0011) (Fig. 1).

#### Effect of AAE on sensitivity

**Fig. 2:**
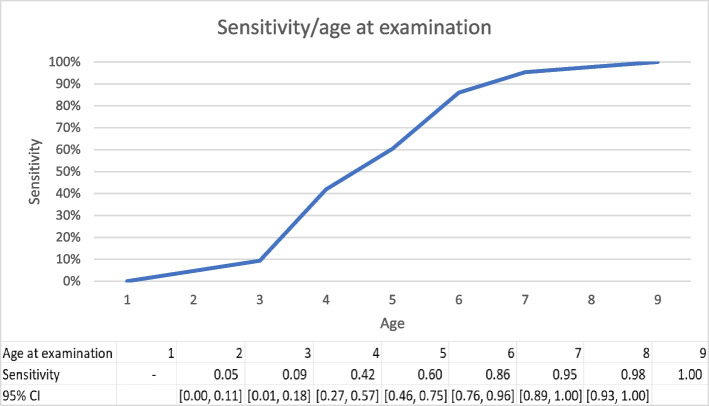
Sensitivity at different ages at examination. Estimated from 43 Havanese in the dataset that had been diagnosed with cataracts within two years after a negative diagnosis

Sensitivity (i.e. the proportion of eventually affected individuals that test positive), was positively correlated with age at examination (Fig. 2). Eye examinations performed at 3 years of age detect only 9% of eventually affected dogs, but at 7 years of age, the likelihood of a correct diagnosis in at-risk individuals increases to 95%.

### Genome wide association analysis

#### Posterior polar cataract

**Fig. 3:**
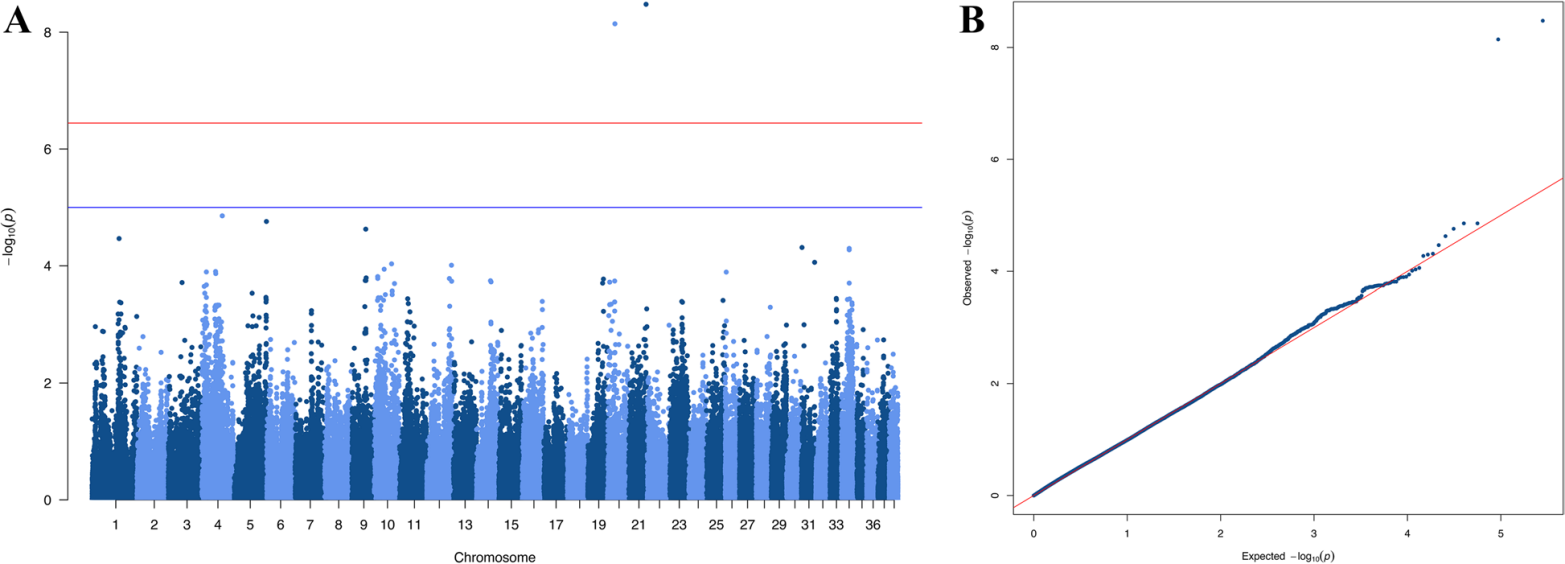
Genome wide association result for posterior polar cataract, including 7 posterior polar cataract cases and 57 controls. The analysis included 140,475 autosomal SNPs that remained after quality control. **A**: Manhattan plot. **B**: Quantile–quantile plot. λ = 1.10

The association analysis of posterior polar cataract revealed two associated SNPs on CFA20 (BICF2S23632983, *p* = 7.2e-09) and CFA21 (BICF2G630640490, *p* = 3.3e-09), that reached genome-wide significance (Fig. 3). Closer inspection of genotypes for BICF2S23632983 showed that the allele associated with posterior polar cataract was present in a heterozygote state in 5/7 cases and only 1/57 controls. For BICF2G630640490, the allele associated with disease was present in a heterozygote state in 6/7 cases and only 2/57 controls.

BICF2S23632983 is positioned at CFA20:20,379,186 (CanFam3.1). Candidate genes in the region include the FOXP1-gene, which is positioned ∼ 60,000 base pairs upstream of the SNP and encodes the Forkhead Box Protein P1. Suzuki-Kerr et. al showed that *Foxp1* is highly expressed in the mural lens during development, and that *Foxp1* knockout mice develop several lens abnormalities, including lens opacities [26]. Lenses from *Foxp1* knockout mice appear normal at birth, but closer inspection reveals increased proliferation and apoptosis of epithelial cells and malformed lens sutures. Two weeks after birth, the knockout mice develop small and opaque lenses with misaligned epithelial cells, abnormal fiber cells, and disorganization of fiber cells in the lens cortex [26]. The essential role of *Foxp1* in normal lens development makes it a highly relevant candidate gene for posterior polar cataract in Havanese.

Another potential candidate gene in the region is the RYBP-gene, positioned ∼700,000 base pairs downstream of BICF2S23632983. RYBP is a zinc finger protein that is essential for normal neurological development [27]. It is highly expressed in the epithelial- and fiber cells of the lens during development, and *Rybp* knockout mice display several eye abnormalities, including malformed lenses [28]. Overexpression of *Rybp* in the lens cause abnormal fiber cell differentiation, altered levels of lens proteins and severe lens opacities [28], which makes it an interesting cataract candidate gene.

BICF2G630640490 is positioned at CFA21:47,286,098 (CanFam3.1), within intron 5–6 of the ANO3 gene. *ANO3* encodes Anoctamin 3 protein, which is a calcium-activated chloride channel [29]. The gene has been found to be associated with dystonia and other neurological disorders [30–32]. Among genes in the region that have previously been associated with cataract development, *LGR4* is positioned ∼800 kb downstream of BICF2G630640490. The LGR4 gene encodes the Leucine Rich Repeat Containing G Protein-Coupled Receptor 4, which is important for normal development of many organs in the body and is a known cataract candidate gene. One study showed that one in four *Lgr4* knockout mice are affected by cataracts, with disorganized and enlarged lens fibres at the cortex of the lens [33]. Insoluble lens proteins and abnormal protein deposits were also observed. The extent of the lens opacities varied between the affected individuals. Another study found that *Lgr4* knockout mice developed lens opacities earlier than wild type mice after exposure to oxidative stressors and had an increased incidence of cataracts, which indicate that *Lgr4* may play an important role in cataract formation [34].

#### Cortical cataract

**Fig. 4:**
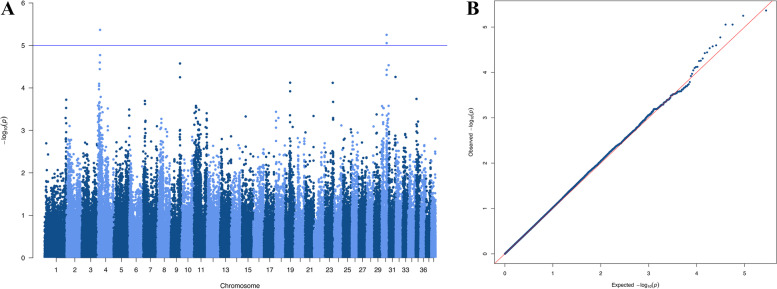
Genome wide association result for cortical cataract, including 27 cortical cataract cases and 57 controls. The analysis included 140,475 autosomal SNPs that remained after quality control. **A**: Manhattan plot. **B**: Quantile–quantile plot. λ = 1.02

The association analysis of cortical cataract did not reveal associations that were significant after Bonferroni correction (Fig. 4), but we observed a suggestive association on CFA4 (chr4_12164500, *p* = 4.3e-06). The top SNP is located ∼1500 base pairs upstream of *ANK3,* and is in LD with multiple SNPs within the gene (*r2* = 0.63–0.76). The ANK3-gene encodes the protein ankyrin-G (also known as ankyrin-3), which functions as a scaffolding protein that links membrane proteins to the intracellular cytoskeleton [35]. One study showed that ankyrin-G is expressed in the lens of mice, particularly in the lateral membranes of the lens epithelium [36]. It plays an obligatory role in normal lens development, and Ank3 knockout mice develop bilateral microphthalmia and cataracts. Suppression of ankyrin-B, which is another member of the ankyrin family, has also been shown to induce cataract formation, through disorganization of lens fibers [37].

Suggestive association was also observed on CFA30, (chr30_28836339, *p* = 5.6e-06; BICF2G630401302, *p* = 8.8e-06; BICF2G630401280, *p* = 8.8e-06) (Fig. 4). The top SNP is positioned at CFA30:28,836,339, within the 3’-UTR of *PCLAF*. The PCLAF-gene encodes a PCNA-binding protein that regulates DNA repair following DNA damage, including damage caused by ultraviolet (UV)-B radiation, which is a well-known cataract risk factor. Chen et. al examined differences in protein- expression and ubiquitination in normal lenses versus lenses subjected to UV-B irradiation, as a model for lens changes in age-related cataracts [38]. Compared to the control lenses, lens epithelial cells that had been subjected to UV-B irradiation showed decreased expression and upregulated ubiquitination of PCNA-binding protein. This indicate that *PCLAF* could influence the development of age-related cataract following UV-B exposure.

Two additional potential candidate genes within the LD-span of the top SNP on CFA30, include *SNX22* and *TPM1. TPM1* is located ∼1.2 Mb downstream of the top SNP and encodes the tropomyosin alpha-1 chain. Shibata et al. 2021 documented the essential role of *Tpm1* in lens fiber differentiation and homeostasis during aging [39]. They showed that *Tpm1* lens-specific knockout mice develop lens opacities, and propose that disturbances in TPM1 regulation could induce cataract formation. *TPM2*, which is a closely related gene, has also been associated with cataract development. One study showed that *Tpm2* knockout mice had an earlier onset and faster progression of cataracts, and that the opacities first appeared in the centre of the anterior cortex [40]. Another study found that the expression of *TPM2* is higher in lenses from humans with severe nuclear cataract, than in lenses from people with milder forms of the disease [41].

*SNX22* is located ∼200 kb upstream of chr30_28836339. One study report that *Snx22* may play a role in the development of congenital cataracts, due to altered expression in cataractous lenses compared to controls [42].

BICF2G630401302 is located within intron 2–3 of *ZNF609,* which encodes the zinc finger protein 609. The gene has been found to influence the development of several eye diseases, but not specifically cataracts [43].

#### Anterior suture line cataract

The association analysis of anterior suture line cataract did not reveal any significantly associated regions (Supplementary Fig. 4). Interestingly, the SNP that showed the second strongest association (BICF2S23512744, *p* = 1.2e-05), was located on CFA6 within in the potential cataract candidate gene, *SDK1* [44], but given the lack of statistical support, the finding should be interpreted with caution.

## Discussion

The study revealed two SNPs, on CFA20 (BICF2S23632983, *p* = 7.2e-09) and CFA21 (BICF2G630640490, *p* = 3.3e-09), that were significantly associated with posterior polar cataract in Havanese. Two candidate genes on CFA20, *FOXP1* and *RYBP*, are located approximately 60 kb and 700 kb from the associated SNPs, respectively, well within a distance in which linkage disequilibrium (LD) is expected [45] and observed (Supplementary Fig. 1). On CFA21, the associated SNP is positioned within *ANO3*, and ∼1 Mb upstream of the highly relevant candidate gene, *LGR4*.

The study identifies two regions of the genome that may harbour genetic variants that are important in the development of posterior polar cataract in Havanese. In humans, posterior polar cataract is considered a distinct type of cataract, which is most often inherited in a dominant fashion [46]. Based on the allele distributions in cases versus controls, a dominant inheritance pattern could potentially fit our results as well, although not with a perfect association. This could be due to modifying genes, incomplete penetrance or incomplete LD between the SNPs and the functional polymorphisms.

We believe the reason we observe single SNPs in the association analysis of posterior polar cataract (rather than a “peak” consisting of multiple SNPs), is the small number of cases, which means strong LD between the marker and the functional variant is necessary to observe a significant association. Closely linked markers with a higher minor allele frequency will be in weaker LD with the functional variant and might not show significant association at this sample size. Although BICF2S23632983 and BICF2G630640490 are not in LD with markers within the candidate genes mentioned, we find relatively long stretches of LD in the region, spanning across the associated variants and the genes discussed (Supplementary Fig. 1).

Sequencing of a selection of cases and controls in the candidate regions is in progress, with the aim of identifying functional polymorphisms in the region. The clear association we find for posterior polar cataract, despite the limited sample size, indicate that there may be one or two mutations that have a major effect on the development of the disease, and that these variants, when identified, may provide a basis for future DNA-testing to support breeding and reduce the prevalence of disease.

The analysis of cortical cataract gave promising results, identifying suggestive associations on CFA30 and CFA4. The top SNP on CFA30 is positioned within the 3’-UTR of *PCLAF*, and within a 1.2 Mb distance of *TPM1* and *SNX22*. The top SNP on CFA4 is located ∼ 1500 base pairs away from *ANK3*, and is in LD with variants within the gene.

The relatively weak association we find in the cortical- and anterior suture line cataract analyses, might indicate a complex mode of inheritance or low heritability. It’s also possible that the lack of observed association in the analysis of anterior suture line cataract is partly contributable to challenges with correct classification, as the pathological changes observed in this form of cataract are subtle.

Continued studies of all three forms of cataracts, using fine mapping, targeted resequencing, or support from WGS-data, may identify functionally associated variants. This would enable the development of DNA-tests, to help breeders avoid breeding Havanese that become affected by the disease. The identification of novel variants that influence cataract development would not only benefit Havanese dogs, but could also provide information that is useful to humans. The low genetic heterogeneity within a dog breed, combined with a spectrum of inherited diseases that are also found in humans, including eye disorders, makes dogs valuable natural models for genetic studies on developmental cataracts.

One aim of this study was to recommend a reasonable minimum age for controls. Our findings support 7 years as reasonable cut-off, as 95% of true positives test positive at this age, compared to 86% one year earlier. Increasing the minimum age for controls to 8 years would improve accuracy of classification further (sensitivity at 8 years of age = 0.98) but could also negatively affect the number of controls available for study. In this study, half of the controls would be excluded using a cut-off age for controls of 8 years.

Even though observed prevalence of cataracts is strongly correlated with AAE between 1 and 8 years, we see a decrease in observed prevalence in dogs aged 9 years or older. A likely explanation is that dogs that are examined at this age is a selection of males that tested negative at age 7 or 8, and are therefore continuously examined because they are still used for breeding.

Until the genetic background for the various forms of cataracts in Havanese is uncovered, selection must be based on phenotype through ECVO eye screening. However, most Havanese in Norway are eye examined at an age (average AAE = 3.44) when it is unlikely to observe clinical changes, even in dogs that are genetically at risk, which will result in a high number of false negatives. We recommend that all Havanese undergo an eye examination prior to breeding, even in situations where the animals are bred at a low age, but emphasize that results from young dogs must be interpreted with caution.

It's important that breeders are aware of the low sensitivity of cataract screening in young dogs. For bitches there are natural limits to how much age at breeding can be increased without compromising welfare, but increasing the proportion of litters sired by older, unaffected males, would likely reduce the risk of producing affected offspring.

## Conclusions

The study identifies two loci on CFA20 and CFA21 that are significantly associated with posterior polar cataract in Havanese. The SNP on CFA20 is located close to two candidate genes, *FOXP1* and *RYBP*, both of which are highly expressed in the lens and are suggested to influence development of lens opacities. The SNP on CFA21 is located within *ANO3,* which has primarily been associated with neurological disease, but is also positioned in the proximity of *LGR4*, which is associated with cataract development in mice. The results suggests that the identified genomic regions may harbour genes with large effect on development of posterior polar cataract in Havanese, possibly in a dominant fashion.

SNPs on CFA4 and CFA30 show putative association with cortical cataract and the top SNP on each chromosome is positioned within, or close to, cataract candidate genes. Polygenetic influence on cortical- and anterior suture line cataract in Havanese is not unlikely.

We also show that age at examination strongly influences sensitivity of cataract screening, and suggest that increasing the average age of breeding animals, and especially of stud dogs, would improve accuracy of selection and genetic improvement.

## Methods

### Population data analyses

Analyses of eye screening statistics, average age at examination (AAE), age at onset (AAO), effect of AAE on prevalence and effect of AAE on sensitivity, were conducted using data from The Norwegian Kennel Clubs open database, “Dogweb” [3]. All available eye screening results for Havanese (*n* = 2581), were included, spanning from year 2003 to 2022. Out of these, 25% (*n* = 649) were performed between 2003 and 2012 and 75% (*n* = 1932) were performed between 2013 and 2022.

#### Age at examination and prevalence

A complete dataset, comprising all available Havanese eye screening records in the NKK database (*n* = 2581), were used to calculate eye screening statistics for years 2003–2022. The same dataset (which includes duplicate individuals when dogs are examined more than once), was used to estimate average age at examination, where “Age at examination” (AAE) was defined as “Year of examination”– “Year of birth”. The full dataset was also used to calculate observed prevalence in groups of dogs based on minimum age at examination.

#### Age at onset and sensitivity

Average age at onset (AAO) was estimated based on a subset of Havanese that had been diagnosed with cataracts within two years after a negative diagnosis (n = 43), by calculating the median of “Age at first positive diagnosis” and “Age at last negative diagnosis”. The same subset of dogs was also used to calculate sensitivity, i.e., the proportion of true positives that test positive, and study how sensitivity is influenced by AAE.

### Genome wide association analysis

#### Dogs

DNA-samples were recruited for the genome wide association study in three ways: 1) DNA-samples stored in our biobank that were originally recruited for other projects [24, 47], matched with Dogweb eye screening records, 2) through online announcements in breed club webpages and online groups for Havanese owners in Norway, Finland, Denmark and Sweden, and 3) through direct inquiries to owners of Havanese that were already registered in the NKK database with an ECVO certificate and met the inclusion criteria. All samples were collected with owners’ consent for use in research.

All DNA-samples recruited specifically for this study were collected using Performagene™ buccal swabs (DNA Genotek Inc), administered by the owner. DNA was extracted following the manufacturer’s recommendations and stored at -20 °C.

Inclusion criteria for cases were purebred Havanese that had been diagnosed with cataracts (cortical, anterior suture line or posterior polar) at any age, either as a part of a routine ECVO screening, or because of clinical signs and subsequent surgery. Inclusion criteria for controls were purebred Havanese that were unaffected by any form of cataracts at 7 years of age or older, confirmed by an ECVO eye certificate. Controls were the same in the three separate association analyses of the different forms of cataracts.

#### Genotyping and association analysis

Dogs were genotyped using Illumina CanineHD 230 K BeadChip. Quality control and filtering was performed using PLINK 1.9 beta 6 [48, 49]. There were 211,256 autosomal variants present prior to filtering. Out of these, 10,389 variants were removed because they were missing in more than 5% of samples (–geno 0.05), 59,545 variants were removed because the minor allele frequency was below 0.05 (–maf 0.05), and 847 variants were removed due to deviation from Hardy Weinberg equilibrium (–hwe 0.001). After quality control, there were 140,475 remaining variants that were included in the association analysis.

Dogs were excluded if more than 5% of genotype data was missing (–mind 0.05). The association analyses included 57 controls; and 27 cortical cataract cases, 23 anterior suture line cataract cases and 7 posterior polar cataract cases; respectively.

High levels of relatedness between individuals and long stretches of linkage disequilibrium are expected in a closed population of purebred dogs [45, 50, 51]. To control for population stratification, the analyses were run using mixed linear models, which included a genetic relationship matrix (GRM) as a random effect. To avoid loss of power from including SNPs in high LD with the candidate SNP in the GRM, we used the “leaving one chromosome out” (MLMA-LOCO) option in GCTA [52, 53], which exclude the chromosome of the candidate SNP from calculation of the GRM. Sex chromosomes were excluded from the association analyses.

Even though the selected models were expected to effectively control population stratification, MDS-plots were created to visually inspect that cases and controls were reasonably distributed in the study population prior to analysis (Supplementary Fig. 2).

Although high levels of linkage disequilibrium between SNPs are expected in purebred dogs [45, 50, 51], we calculated a conservative Bonferroni-corrected genome wide significance level of 3.6e-07, corrected for the number of all autosomal variants that remained after quality control (*n* = 140,475). The threshold for suggestive significance was set at the default *p*-value of 1.0e-05.

Manhattan plots and QQ-plots were created in R version 4.2.1 [54], using the R-package qqman version 0.1.8 [55].

#### Mapping of linked genes

Genes near the top SNPs (posterior polar and anterior suture line: 1 Mb upstream and downstream, cortical: 0.5 Mb upstream and downstream) were inspected and checked for any relevant involvement in cataract development using PubMed® [56]. Additionally, a larger region (≈ ± 2 Mb) surrounding the top SNPs were controlled against the online chromosome map and reference database for inherited and age-related cataracts, Cat-Map [57], after conversion of coordinates from CanFam3.1 to GRCh38 using the LiftOver-function in the UCSC genome browser [58].

## Supplementary Information

**Additional file 1: Supplementary figure 1.** A schematic illustration of LD-stretches in the associated regions on (A) CFA20 and (B) CFA21, showing LD between selected SNPs that span across the associated variants (orange outline) and potential candidate genes (orange). (Additional SNPs show similar values but are not shown).** Supplementary figure 2.** MDS-plots for the (A) posterior polar cataract and (B) cortical cataract datasets, showing that both cases and controls are represented in each cluster for both phenotypes. Cases = red, controls = blue, unknown status (excluded from the association analysis) = grey.** Supplementary figure 3.** Regional Manhattan plots, spanning +/- 2 Mb of the top SNP on each chromosome of the association analysis of posterior polar cataract (A+B) and cortical cataracts (C+D). A: CFA20, B: CFA21, C: CFA4, D: CFA30.** Supplementary figure 4.** Genome wide association result for anterior suture line cataract, including 23 anterior suture line cataract cases and 57 controls. The analysis included 140475 autosomal SNPs that remained after quality control. A: Manhattan plot. B: Quantile-quantile plot. λ = 1.03.

## Data Availability

Analyses of eye screening statistics, average age at examination (AAE), age at onset (AAO), effect of AAE on prevalence and effect of AAE on sensitivity, were conducted using data from The Norwegian Kennel Clubs database, which is publicly available.

## Abbreviations

AAE: Age at examination, here defined as “year of examination” – “year of birth”
AAO: Age at onset
CFA: Canine chromosome
ECVO: European College of Veterinary Ophthalmologists
GCTA: Genome-wide Complex Trait Analysis
GRCh38: Genome Research Consortium human build 38
GRM: Genomic relationship matrix
GWAS: Genome wide association study
LD: Linkage disequilibrium
SNP: Single nucleotide polymorphism
