# Supplementary material for: Cataracts in Havanese: genome wide association study reveals two loci associated with posterior polar cataract
Source: Canine Med Genet. 2023 Apr 28;10:5. doi: 10.1186/s40575-023-00127-y (PMC10142750; doi:10.1186/s40575-023-00127-y)
Supplement: Supplementary file 1 — Additional file 1: Supplementary figure 1. A schematic illustration of LD-stretches in the associated regions on (A) CFA20 and (B) CFA21, showing LD between selected SNPs that span across the associated variants (orange outline) and potential candidate genes (orange). (Additional SNPs show similar values but are not shown). Supplementary figure 2. MDS-plots for the (A) posterior polar cataract and (B) cortical cataract datasets, showing that both cases and controls are represented in each cluster for both phenotypes. Cases = red, controls = blue, unknown status (excluded from the association analysis) = grey. Supplementary figure 3. Regional Manhattan plots, spanning +/- 2 Mb of the top SNP on each chromosome of the association analysis of posterior polar cataract (A+B) and cortical cataracts (C+D). A: CFA20, B: CFA21, C: CFA4, D: CFA30. Supplementary figure 4. Genome wide association result for anterior suture line cataract, including 23 anterior suture line cataract cases and 57 controls. The analysis included 140475 autosomal SNPs that remained after quality control. A: Manhattan plot. B: Quantile-quantile plot. λ = 1.03. [file 40575_2023_127_MOESM1_ESM.pdf]

Supplementary materials

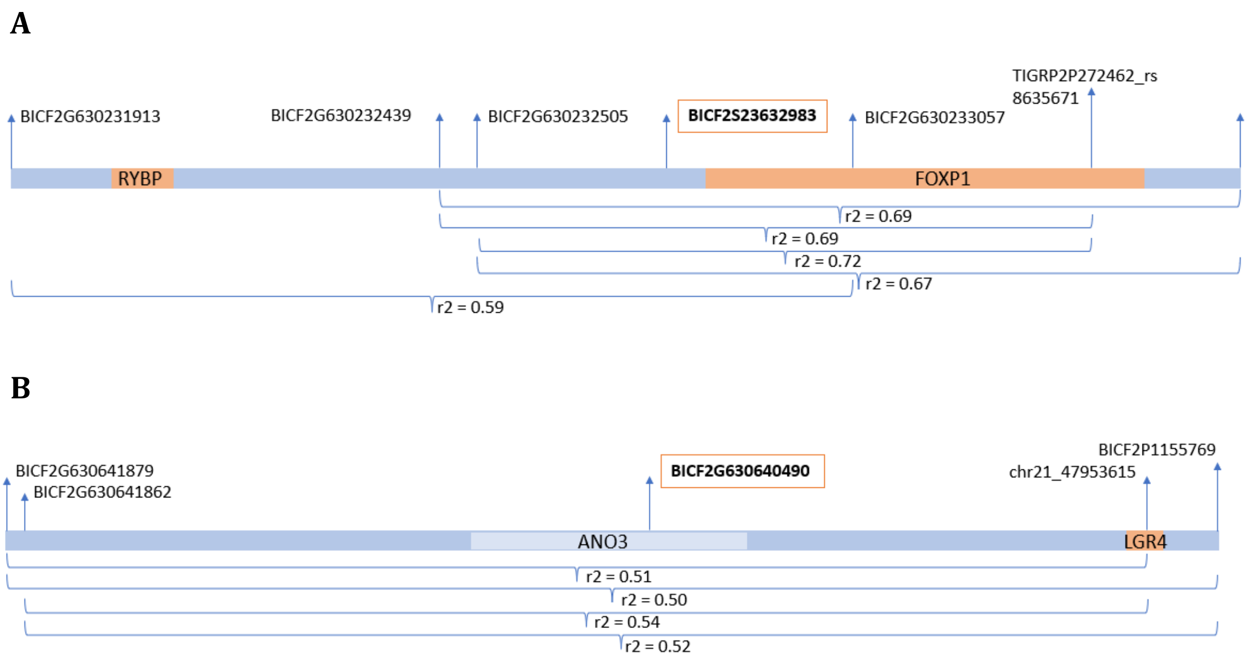

Supplementary figure 1: A schematic illustration of LD-stretches in the associated regions on (A) CFA20 and (B) CFA21, showing LD between selected SNPs that span across the associated variants (orange outline) and potential candidate genes (orange). (Additional SNPs show similar values but are not shown).

A

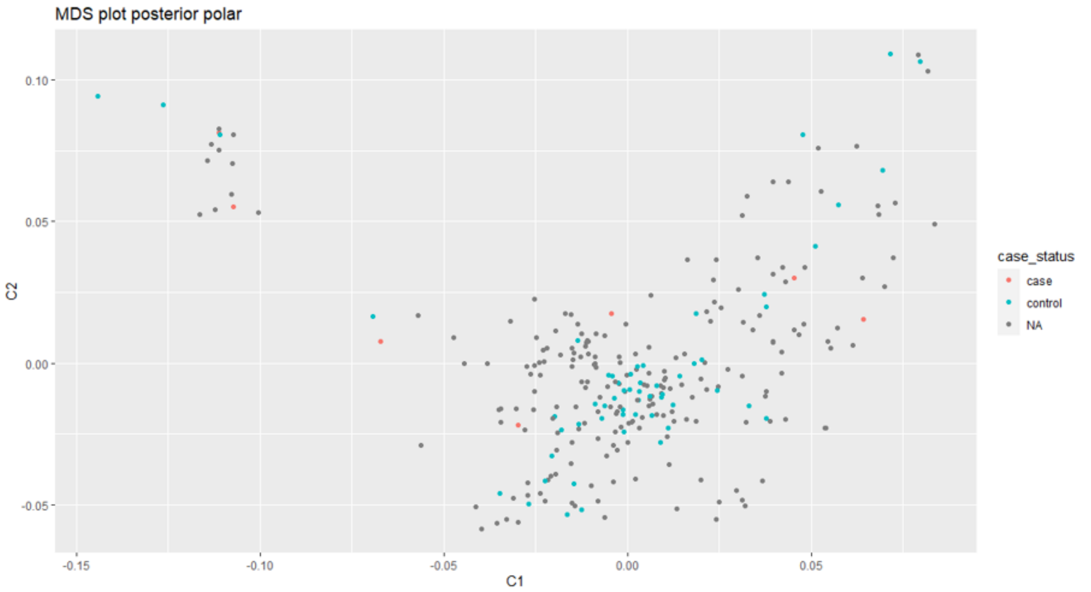

B

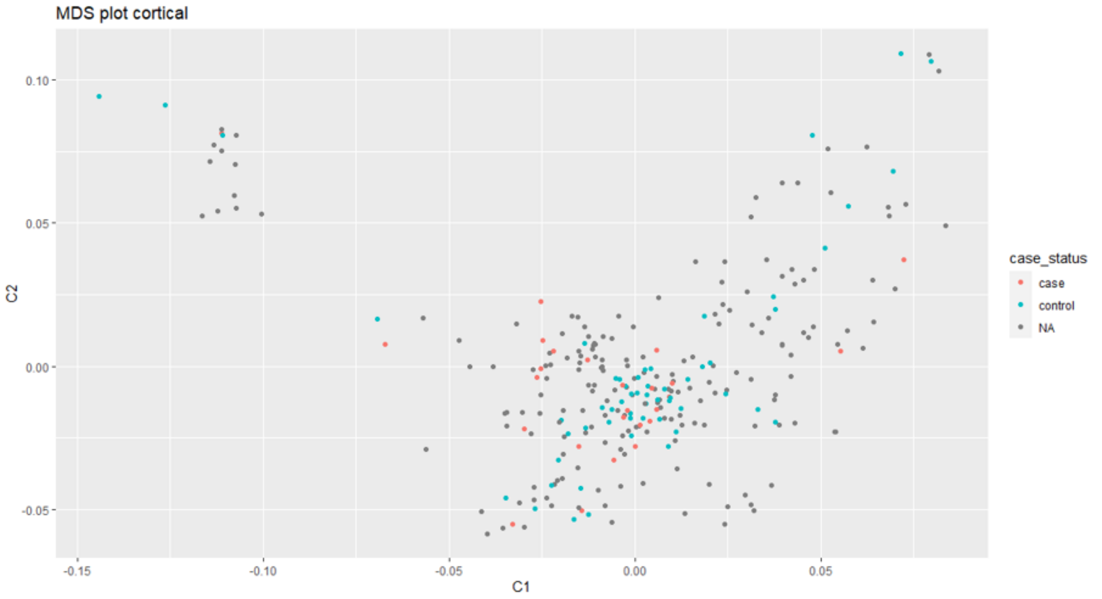

Supplementary figure 2: MDS-plots for the (A) posterior polar cataract and (B) cortical cataract datasets, showing that both cases and controls are represented in each cluster for both phenotypes. Cases = red, controls = blue, unknown status (excluded from the association analysis) = grey.

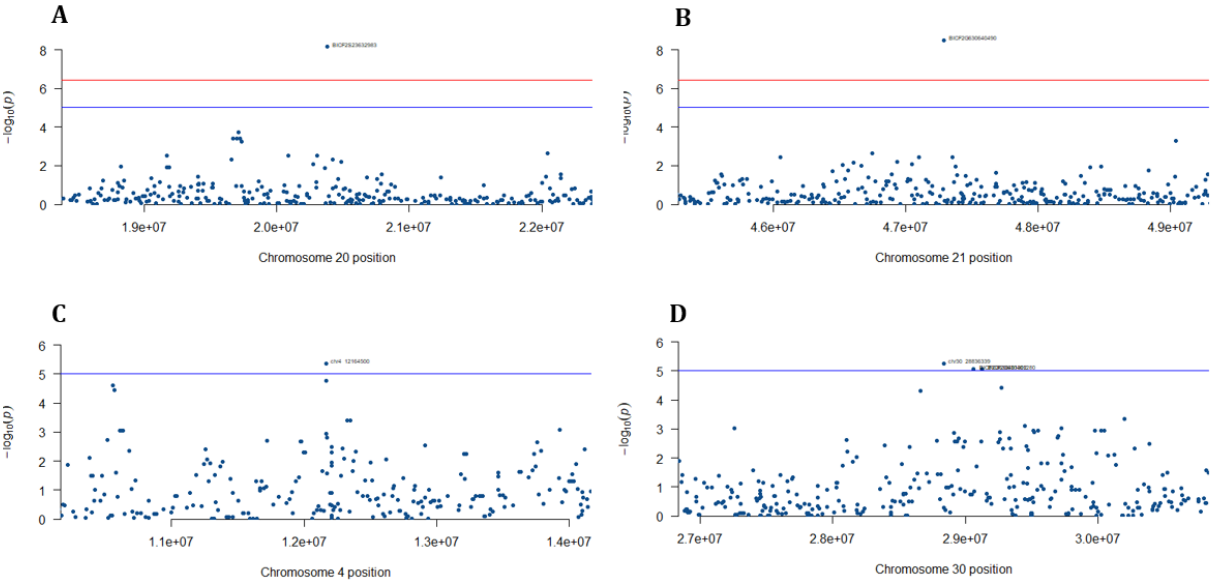

Supplementary figure 3: Regional Manhattan plots, spanning +/- 2 Mb of the top SNP on each chromosome of the association analysis of posterior polar cataract (A+B) and cortical cataracts (C+D). A: CFA20, B: CFA21, C: CFA4, D: CFA30.

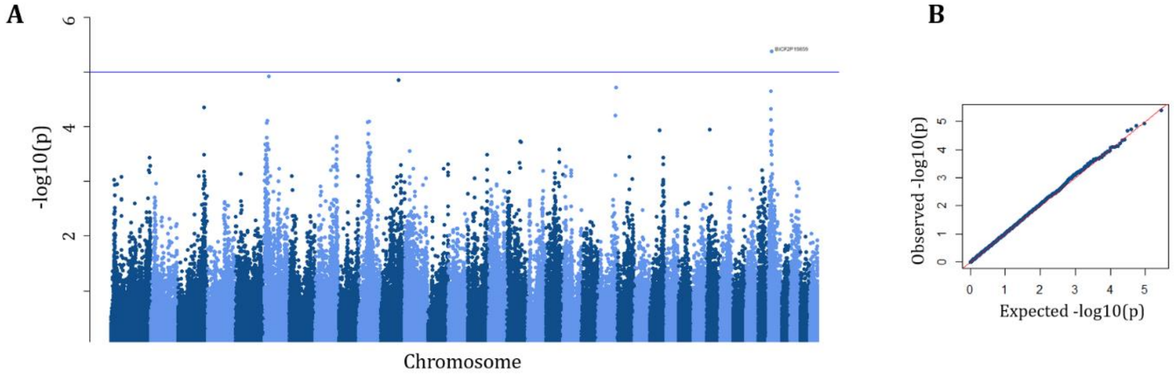

Supplementary figure 4: Genome wide association result for anterior suture line cataract, including 23 anterior suture line cataract cases and 57 controls. The analysis included 140475 autosomal SNPs that remained after quality control. A: Manhattan plot. B: Quantile-quantile plot. λ = 1.03.
